# Which Matters More in Incidental Category Learning: Edge-Based Versus Surface-Based Features

**DOI:** 10.3389/fpsyg.2019.00183

**Published:** 2019-02-07

**Authors:** Xiaoyan Zhou, Qiufang Fu, Michael Rose, Yuqi Sun

**Affiliations:** ^1^ State Key Laboratory of Brain and Cognitive Science, Institute of Psychology, Chinese Academy of Sciences, Beijing, China; ^2^ Department of Psychology, University of Chinese Academy of Sciences, Beijing, China; ^3^ NeuroImage Nord, Department for Systems Neuroscience, University Medical Center Hamburg Eppendorf, Hamburg, Germany

**Keywords:** category learning, incidental category learning, the prototype distortion task, edge-based features, surface-based features

## Abstract

Although many researches have shown that edge-based information is more important than surface-based information in object recognition, it remains unclear whether edge-based features play a more crucial role than surface-based features in category learning. To address this issue, a modified prototype distortion task was adopted in the present study, in which each category was defined by a rule or a similarity about either the edge-based features (i.e., contours or shapes) or the corresponding surface-based features (i.e., color and textures). The results of Experiments 1 and 2 showed that when the category was defined by a rule, the performance was significantly better in the edge-based condition than in the surface-based condition in the testing phase, and increasing the defined dimensions enhanced rather than reduced performance in the edge-based condition but not in the surface-based condition. The results of Experiment 3 showed that when each category was defined by a similarity, there was also a larger learning effect when the category was defined by edge-based dimensions than by surface-based dimensions in the testing phase. The current study is the first to provide convergent evidence that the edge-based information matters more than surface-based information in incidental category learning.

## Introduction

Edge-based information (e.g., line, shape, and contour) always appears at boundaries to separate the object from the background and could be preserved in line drawings. Surface characteristics (e.g., color, brightness, and texture) always define the physical description of the stimulus and are included in color photographs rather than line drawings. Although it remains controversial whether edge-based information plays a primary role in object recognition (Delorme et al., 2000; Gegenfurtner and Rieger, 2000; Tanaka et al., 2001; Bramão et al., 2011; Moreno-Martínez and Rodríguez-Rojo, 2015; Rokszin et al., 2015), a substantial number of studies have shown that edge-based representations are sufficient for object or scene recognition, whereas surface characteristics are less efficient routes for accessing the memorial representation (Biederman, 1987; Biederman and Ju, 1988; Diesendruck and Bloom, 2003; Elder and Velisavljević, 2009; Walther et al., 2011; Rokszin et al., 2015; Fu et al., 2016). For example, it has been found that the mean reaction times and error rates in the naming task and the verification task were identical for common objects of line drawings and color pictures when the images were presented briefly (Biederman, 1987; Biederman and Ju, 1988), and there were no significant differences in the performance for rapid natural scene categorization of color and gray images (Delorme et al., 2000, 2010). It has been also demonstrated that surface-based information, such as color, could facilitate visual processing only when shape information is degraded (Tanaka and Presnell, 1999) or when there was no time limit for the stimulus presentation (Wurm et al., 1993).

If category representation consists primarily of edge-based information, as novel representations of different categories are formed during category learning, one would expect that edge-based and surface-based information might also have different roles in category learning. Most studies on category learning focused on what type of category representations are formed (Ashby and Maddox, 2005, 2011; Ashby and O’Brien, 2005; Richler and Palmeri, 2014; Serre, 2016) and how they are learned and generalized (Nosofsky and Zaki, 2002; Ashby et al., 2003; Maddox and Ashby, 2004; Casale and Ashby, 2008; Seger and Miller, 2010; Ell et al., 2017). However, to the best of our knowledge, no study has addressed the roles of edge-based and surface-based features in category learning, although examining this issue has important implications for computational models and theories of category learning.

It has been demonstrated that the category could be represented by a rule or a similarity in category learning. The rule-based theory posits that the category representation consists of a verbal rule of prominent features (Maddox et al., 2003; Maddox and Ashby, 2004; Ashby and Maddox, 2005, 2011; Carpenter et al., 2016; Ashby and Valentin, 2017), which specifies definitively whether an object or an event is of a particular sort or not (Shanks, 1995, p. 152). Different from the rule-based theories, the similarity-based theory posits that people form a summary representation in the form of prototypes crucial to the category representation (Knowlton and Squire, 1993; Reber et al., 1998a,b; Reed et al., 1999; Smith, 2002; Smith and Minda, 2002; Bozoki et al., 2006; Homa et al., 2011), or store category members as individuated memory representations that constitute the category representation (e.g., Nosofsky and Zaki, 2002; Zaki and Nosofsky, 2004, 2007; Tunney and Fernie, 2012). The models of the similarity-based theory hold that the categorization of novel stimuli is achieved by comparing them with the category representation. However, neither theory takes into account the role of edge-based and surface-based features in category learning.

To address this issue, a modified prototype distortion task was adopted in the present study. Typically, in the prototype distortion task, the category is created by first defining a category prototype and then creating the category members by randomly distorting the prototype (Knowlton and Squire, 1993; Reed et al., 1999; Bozoki et al., 2006; Nosofsky et al., 2012; Gorlick and Maddox, 2013; Heindel et al., 2013). All dimensions of the prototype are relevant to the category membership. In the training phase, participants were presented with low or high distortions of the prototype. They were asked to give likability ratings or memorize them, which have no reference to subsequent testing. Then, in the testing phase, they were informed that the stimuli presented in the training phase belong to one category, and they were asked to judge whether novel items, i.e., the unseen prototypes and different types of distortions, belong to that category in the training phase. Generally, the results showed that participants endorsed the previous unseen prototype as belonging to the category with the highest probability, followed by the low-level distortions, high-level distortions, and random patterns, which is termed as “prototype gradient effect” (Knowlton and Squire, 1993; Bozoki et al., 2006; Homa et al., 2011; Zannino et al., 2012). It is argued that the prototype gradient effect indicated that category knowledge might be acquired by abstracting information across encounters with examples in the training, i.e., in the form of information about prototype (Knowlton and Squire, 1993). As no trial-by-trial feedback is provided in the training and testing phase, this incidental task is more typical of the real-world learning situations (Love, 2002, 2003).

To investigate the role of different types of features in category learning, we divided stimulus features into edge-based features and surface-based features. Each category was defined by either edge-based features or surface-based features. Only dimensions of the defined features were relevant to the category membership. In Experiment 1, to ensure that participants could learn the categories, the category was defined by a three-feature-based rule. That is, the category members presented in the training phase shared same features of the defined dimensions. In Experiment 2, we increased the defined dimensions from three to four to further explore whether participants would express implicit knowledge under this incidental situation and whether they would have higher accuracy in the edge-based condition than in the surface-based condition. In Experiment 3, to increase the learning difficulty, each category was defined by a five-feature-based similarity. Stimuli with four defined features identical to the prototypes, i.e., low distortions, were presented in the training phase. That is, no same defined feature was shared by all stimuli in the training phase, which was consistent with the typical prototype distortion task. If edge-based features are more important than surface-based features in the formation of the categorical representations, we would expect that the classification accuracy in the testing phase would be higher when the category was defined by edge-based features than by surface-based features whenever the category is defined by a rule or a similarity; otherwise, the classification accuracy in the testing phase would be higher when the category was defined by surface-based features than by edge-based features, or there would be no difference in the learning effects between the two conditions.

## Experiment 1

**Figure 1:**
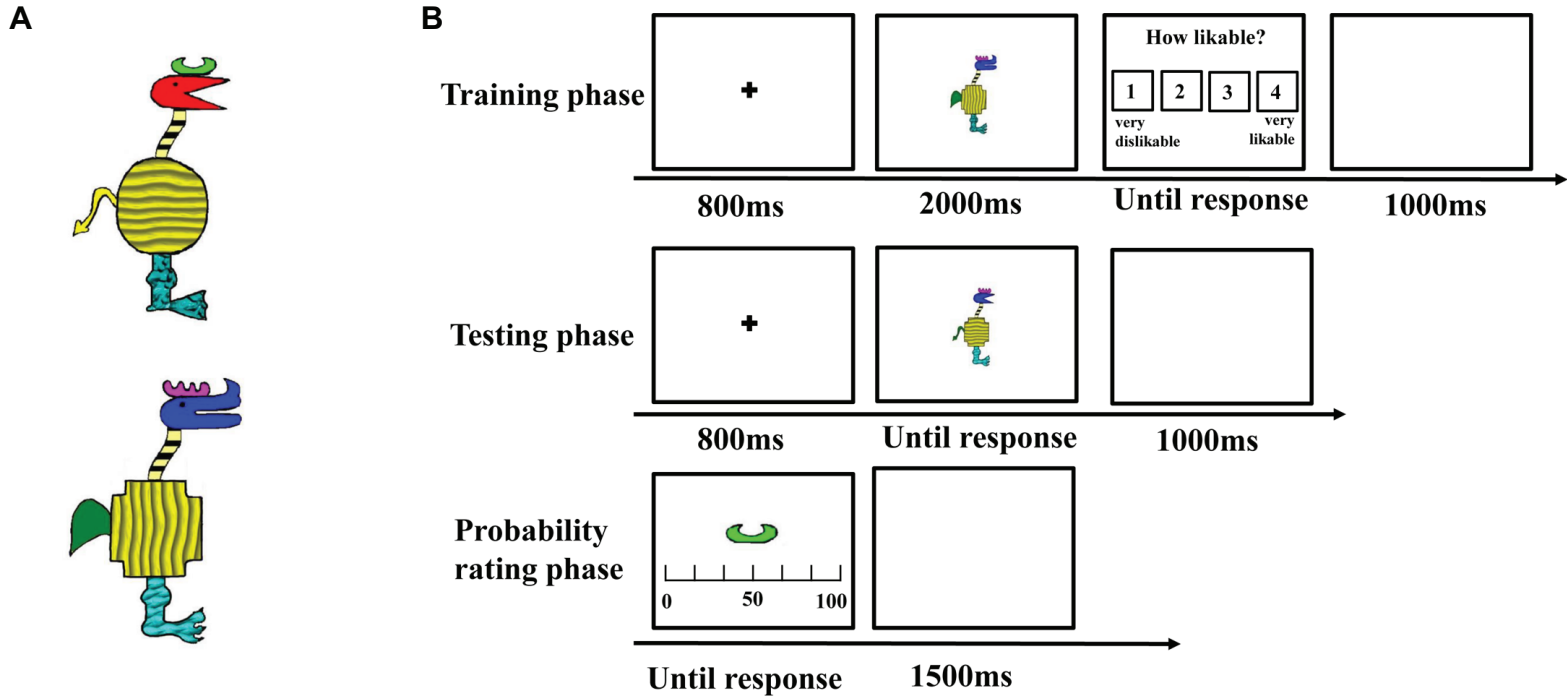
Stimuli and procedure. **(A)** Examples of stimuli with different features on 10 dimensions and **(B)** the trial procedure of the training, the testing and the probability rating phases in Experiment 1.

We adopted stimuli from the study of Gorlick and Maddox (2013) in which cartoon animals were constructed from 10 binary dimensions and each dimension has two features. For example, the shape of the horn can be like a comb or the moon; the shape of the head can be acutilingual or lamellirostral. To compare the roles of edge-based features and surface-based features in category learning, five edge-based dimensions, including the shapes of the horn, head, body, tail, and leg, were maintained, and five corresponding surface-based dimensions, including the color of the horn, head, tail, and the texture of the body and leg, were added. As a result, the current stimuli varied along 10 binary dimensions, with five edge-based dimensions (i.e., contours or shapes), and five surface-based dimensions (i.e., colors or textures) as shown in Figure 1A. To ensure that the category could be learned, each category was defined by a three-feature-based rule in Experiment 1. That is, the category members all possess the same features in the three defined dimensions but different features in the other seven dimensions. For example, in the edge-based condition, category members would be those with a comb horn, a paw-shaped leg, and a short and round tail. In the surface-based condition, category members would be those with a violet horn, a cuspidal leg, and a green tail.

### Method

#### Participants

Forty-two university students (20 females and 22 males) voluntarily participated in the experiment. They were randomly assigned to one of the two conditions. There were 20 (9 females; mean age = 21.8 years, *SD* = 2.12) in the edge-based condition and 22 (11 females; mean age = 22.05 years, *SD* = 4.74) in the surface-based condition. All participants reported normal or corrected to normal vision and were paid for their attendance. All experiments were approved by the Institutional Review Board of the Institute of Psychology, Chinese Academy of Sciences. Data from one participant in the edge-based condition and data from two participants in the surface-based condition were excluded from further analysis because their reaction times or accuracies were larger than the means plus two standard deviations.

#### Materials

**Figure 2:**
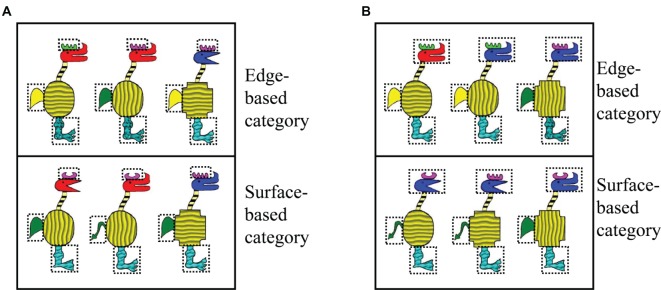
Examples of category members in Experiments 1 and 2. **(A)** Examples of category members in Experiment 1 and **(B)** examples of category members in Experiment 2. The three defined features in Experiment 1 and the four features in Experiment 2 were marked with dotted boxes.

The stimuli were cartoon animals that varied along 10 binary dimensions, with five edge-based dimensions, including the shape of the horn, head, body, tail, and leg, and five surface-based dimensions, including the color of the horn, head, tail, and the texture of the body and leg. Each dimension has two features. Each category member was created by a rule of three fixed features in three defined dimensions. For the edge-based condition, the category members were defined by a rule of three fixed features in regard to the shape of the horn, tail, and leg; correspondingly, for the surface-based condition, the category members were defined by a rule of three fixed features in regard to the color of the horn and tail and the texture of the leg (see Figure 2A, the defined features were marked with dotted boxes). The features of the three defined dimensions were fixed, and the features of the other seven dimensions could change randomly. Thus, there were a total of 128 category members in each condition. Sixty-eight category members were used in the training phase, and the remaining 60 members were used in the testing phase. In order to form the stimuli that did not belong to the category, one defined feature was changed for half of the 60 category members and two defined features were changed for the other half, while the other features were maintained. Therefore, there were three types of testing stimuli according to the features of the defined dimensions: stimuli with one, two, or three defined features in the three defined dimensions.

#### Design and Procedure

The experimental design was a one factor (defined dimensions: edge-based vs. surface-based) between-subject design. Each condition included a training phase, a testing phase, and a probability rating phase (as shown in Figure 1B).

##### Training Phase

The stimuli were presented on a 17-inch CRT monitor and subtended a visual angle of less than 12° (see Nosofsky et al., 2012). Each trial began with a fixation cross at the center for 800 ms, and then, a stimulus was presented for 2000 ms. Participants were instructed to carefully observe each stimulus and rate how likeable it was from 1 (very unlikeable) to 4 (very likeable) after it disappeared on each trial. The inter-trial interval was 1,000 ms. There were 20 trials in the training phase. The stimuli were randomly selected from 68 category members for each participant.

##### Testing Phase

After the training phase, participants were informed that all of the stimuli they had seen in the training phase belonged to one category and they were asked to judge whether the novel stimuli belonged to the category or not in the testing phase. On each trial, a stimulus appeared and remained on the screen until participants made a response by pressing one of two keys with labels “yes” and “no.” After their response, the stimulus disappeared without feedback. The next trial was initiated following a 1,000-ms inter-trial interval. There were 120 trials in the testing phase, in which 60 stimuli belonged to the category and 60 stimuli did not belong to the category (including 30 stimuli with one defined feature changed and 30 stimuli with two defined features changed).

##### Probability Rating Phase

In the final part, each defined dimension with different features such as comb-like horn in blue was presented, and participants were asked to report the probability that the stimuli including the feature of the dimension belonged to the category in the training phase, i.e., the probability for the “yes” response. Participants indicated the probability on a continuous sliding scale from 0 to 100, where 0 = definitely no, 50 = equally likely to be yes or no, and 100 = definitely yes. There were 12 trials in the probability rating phase as there were three defined dimensions.

### Results

#### Accuracy in the Testing Phase

**Figure 3:**
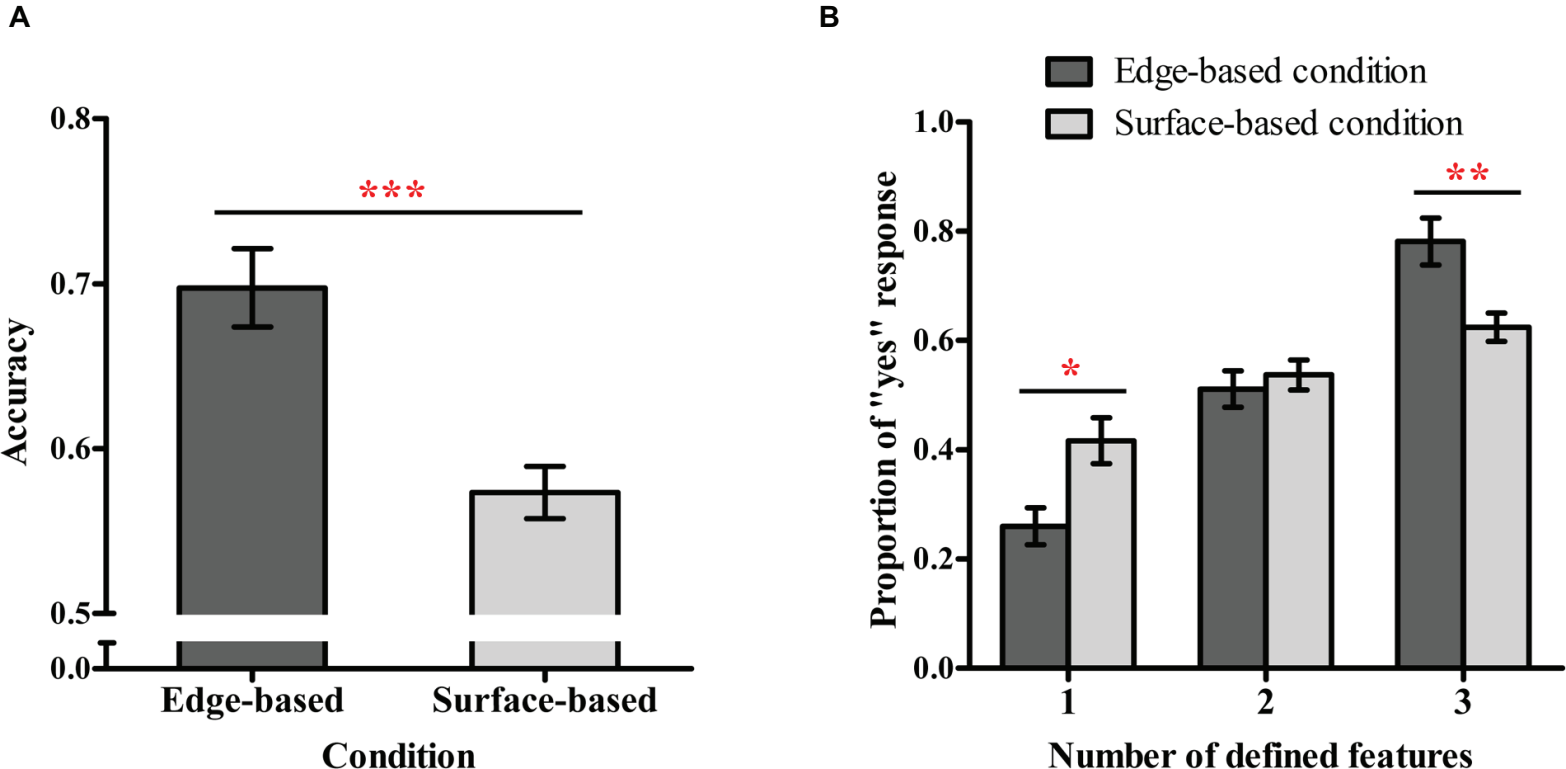
Performance in the testing phase in Experiment 1. **(A)** Accuracy in the edge-based and surface-based conditions. **(B)** Proportions of “yes” responses for stimuli with different numbers of defined features in the edge-based and surface-based conditions. Error bars depict standard errors. *p < 0.05, **p < 0.01, ***p < 0.001.

Figure 3A shows the accuracy for each condition in Experiment 1. A one-sample *t* test was used to examine whether participants could learn the category in incidental category learning. The result revealed that participants in both conditions performed significantly above chance (0.50), *t*_contour_ (18) = 7.94, *p* < 0.001, Cohen’s *d_z_* = 1.82; *t*_surface_ (19) = 4.65, *p* < 0.001, Cohen’s *d_z_ =* 1.04, indicating that all participants learned how to classify the stimuli. To explore the role of different types of features in incidental category learning, an independent-sample *t* test was conducted. The result showed that the accuracy in the edge-based condition (*M* = 0.70, *SD* = 0.11) was significantly higher than in the surface-based condition (*M* = 0.57, *SD* = 0.07), *t*(37) = 4.28, *p* < 0.001, Cohen’s *d* = 1.42. Thus, consistent with our prediction, participants in the edge-based condition performed better than those in the surface-based condition.

#### Categorization Proportions in the Testing Phase

Figure 3B shows the proportions of “yes” responses for different types of testing stimuli in the two conditions. To examine whether the number of the defined features included in the testing stimuli influenced classification performance, a 2 (condition: edge-based vs. surface-based) × 3 (the defined features: 1 vs. 2 vs. 3) mixed ANOVA on the proportion of “yes” responses was conducted. There was a significant main effect of defined features, *F*(2, 74) = 72.86, *p* < 0.001, $\eta_{p}^{2}$ = 0.66, which was modulated by the condition, *F*(2, 74) = 13.97, *p* < 0.001, $\eta_{p}^{2}$ = 0.27. The main effect of condition was not significant, *F*(1, 37) = 0.12, *p* = 0.74. Simple effect analysis showed that for stimuli with one defined feature, the proportion of “yes” responses (i.e., the false alarm rate) was significantly lower in the edge-based condition than in the surface-based condition, *F*(1, 37) = 7.18, *p* < 0.05, $\eta_{p}^{2}$ = 0.16; for stimuli with three defined features, the proportion of “yes” responses (i.e., the hit rate) was significantly higher in the edge-based condition than in the surface-based condition, *F*(1, 37) = 14.18, *p* < 0.01, $\eta_{p}^{2}$ = 0.28. That is, compared with the surface-based condition, participants in the edge-based condition could more correctly reject non-category members and accept category members. In addition, the proportion of “yes” responses significantly increased with the number of defined features in both conditions (both *p*s < 0.001), suggesting that participants learned to combine the features of three dimensions to classify the novel stimuli.

#### Probability Ratings

**Figure 4:**
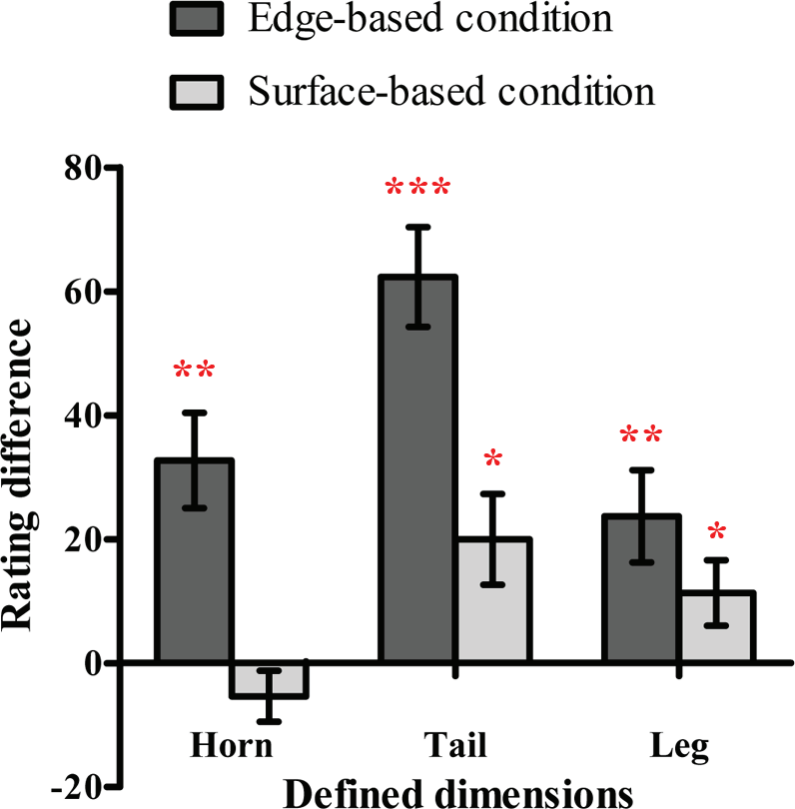
The difference scores of the probability rating for defined dimensions in the edge-based and surface-based conditions in Experiment 1. Error bars depict standard errors. *p < 0.05, **p < 0.01, ***p < 0.001.

To examine whether participants were aware of the relation between the defined features and the category membership, we first calculated the average rating when the defined dimension had or did not have the defined feature separately and then obtained the difference ratings between them (as shown in Figure 4). If the difference rating was significantly above zero, it would indicate that participants might be aware that the defined features were related to the category membership, and vice versa. The one-sample *t* test revealed that the difference ratings of the shape of the horn, tail, and leg in the edge-based condition were significantly above zero [horn shape: *t*(18) = 3.93, *p* < 0.01, Cohen’s *d_z_* = 0.90; tail shape: *t*(18) = 7.31, *p* < 0.001, Cohen’s *d_z_* = 1.68; leg shape: *t*(18) = 2.93, *p* < 0.01, Cohen’s *d_z_* = 0.67]. The difference ratings of the color of the tail and the texture of the leg in the surface-based condition were significantly above zero [tail color: *t*(19) = 2.73, *p* < 0.05, Cohen’s *d_z_* = 0.61; leg texture: *t*(19) = 2.14, *p* < 0.05, Cohen’s *d_z_* = 0.48]. The results indicated that participants in both conditions might be partially aware of the relation between the defined features and the category membership.

In order to figure out whether participants indeed utilized the knowledge they reported in the probability ratings to make classifications in the testing phase, the accuracy was regressed on the significant rating differences of the defined dimensions. For the edge-based condition, the rating difference of the horn shape, tail shape, and leg shape did significantly predict the accuracy in the testing phase, *F*(3, 15) = 3.98, *p* < 0.05, with an adjusted *R* ^2^ of 0.32. For the surface-based condition, the rating difference of the tail color and leg texture did significantly predict the accuracy in the testing phase, *F*(2, 17) = 6.53, *p* < 0.01, with an adjusted *R* ^2^ of 0.37. The results indicated that the awareness scores in the probability rating phase could account for accuracy in the classification task in the testing phase in both conditions.

### Discussion

The results of Experiment 1 showed that participants performed significantly above chance in each condition, indicating that they learned how to classify the stimuli incidentally. Importantly, consistent with our expectation, participants in the edge-based condition performed better than those in the surface-based condition, and they could more correctly reject non-category members and accept category members. The results suggest that the edge-based information is more important than the surface-based information in incidental category learning. Moreover, in both conditions, the “yes” responses gradually increased with the numbers of defined features, which were similar to the typical results of the “prototypical gradient” in previous studies (Knowlton and Squire, 1993; Reed et al., 1999; Bozoki et al., 2006). On the one hand, the results indicate that the features of the three defined dimensions can be combined to make correct classification; on the other hand, the results support the notion that participants might form the category representation in the form of a prototype that includes the features of three defined dimensions. Interestingly, although people learned the category incidentally, the awareness scores in the probability rating phase indicated that they might be partially aware of the relation between the defined features and the category membership and they could use these knowledge to make classification in the testing phase.

## Experiment 2

It has been found that increasing the complexity of a defined rule makes it less likely for observers to learn a category through an explicit reasoning process (Ashby and Casale, 2003). Thus, in Experiment 2, we increased the defined dimensions from three to four to further explore whether participants would express implicit knowledge under this incidental situation and whether they would have higher accuracy in the edge-based condition than in the surface-based condition.

### Method

#### Participants

Forty-seven university students (26 females and 21 males) voluntarily participated in the experiment. They were randomly assigned to one of the two conditions. There were 24 (12 females; mean age = 22.75 years, *SD* = 2.29) in the edge-based condition, and 23 (14 females; mean age = 21.82 years, *SD* = 4.50) in the surface-based condition. All participants reported normal or corrected to normal vision and were paid for their attendance. Data from one participant in the edge-based condition and one participant in the surface-based condition were excluded from further analysis because their accuracy was lower or higher than the mean accuracy minus or plus two standard deviations.

#### Materials

The stimuli were similar to Experiment 1 except that the category was defined by the features of four defined dimensions (as shown in Figure 2B, the four defined features were marked with dotted boxes). For the edge-based condition, the defined dimensions were the shape of the horn, tail, leg, and head. For the surface-based condition, the defined dimensions were the color of the horn, tail, and head and the texture of the leg. The features of the defined dimensions were fixed, while the features of the other would change randomly. As a result, there were 64 category members in each condition. Twenty members were used in the training phase, and the other 44 members were used in the testing phase. In order to form the stimuli that did not belong to the category, we changed one or two or three of the defined features of the 44 stimuli, respectively, while the features of the other dimensions remained unchanged. There were four types of testing stimuli according to the number of defined features for the 44 examples, for a total of 176 in each condition.

#### Design and Procedure

The design and procedure were identical to Experiment 1 except that there were 176 trials in the testing phase and 16 trials in the probability rating phase.

### Results

#### Accuracy in the Testing Phase

**Figure 5:**
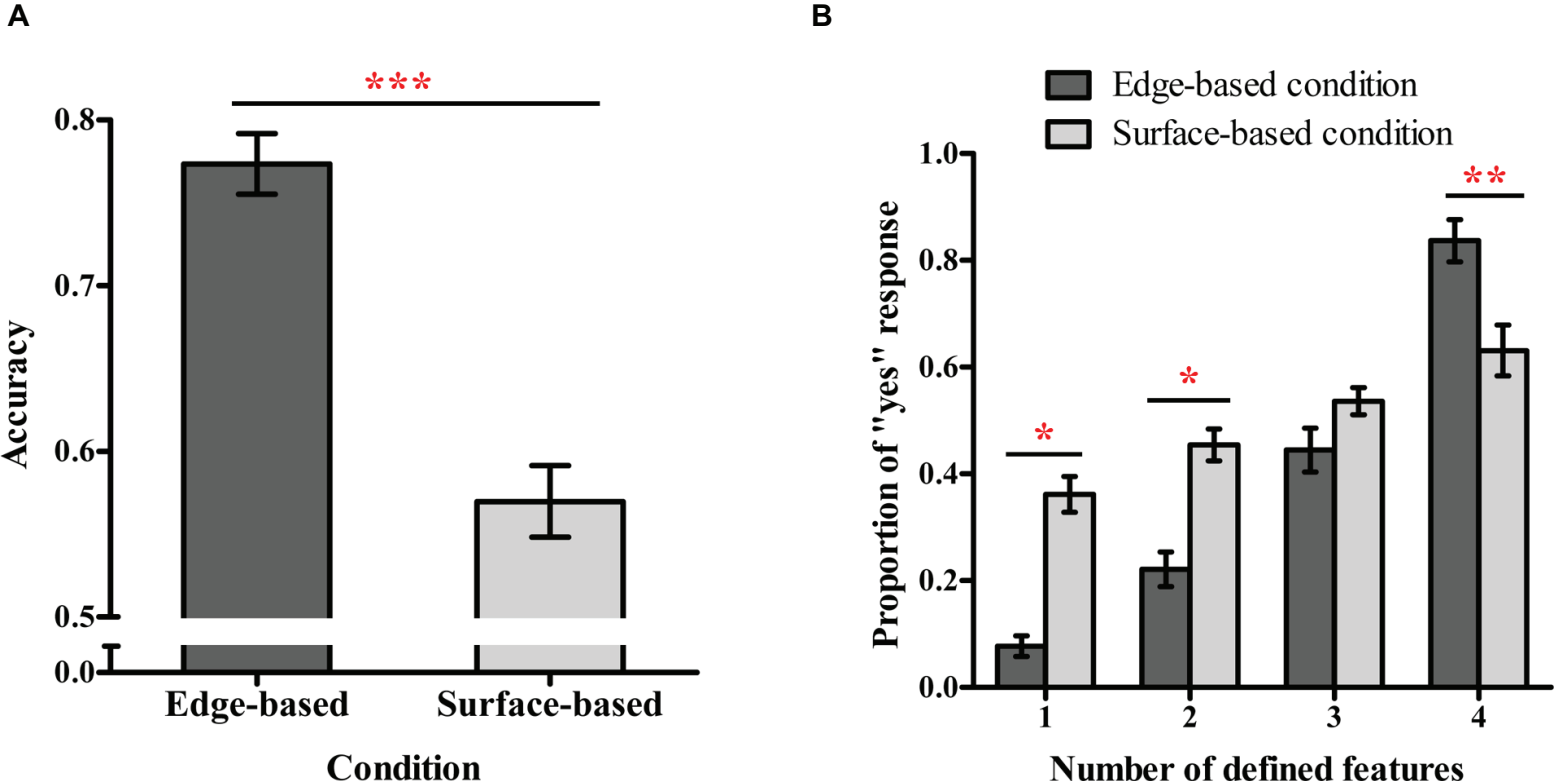
Performance in the testing phase in Experiment 2. **(A)** Accuracy in the edge-based and surface-based conditions. **(B)** Proportion of “yes” responses for stimuli with different numbers of defined features in the edge-based and surface-based conditions. Error bars depict standard errors. *p < 0.05, **p < 0.01, ***p < 0.001.

Figure 5A shows the accuracy for each condition in Experiment 2. As in Experiment 1, in order to examine whether a participant could learn the category in incidental category learning, we first used a one-sample *t* test to compare the performance in each condition with chance (0.50). The result revealed that participants in both the edge-based condition and the surface-based condition learned how to classify the stimuli [edge-based: *M* = 0.77, *SD* = 0.09, *t*(22) = 14.96, *p* < 0.001, Cohen’s *d_z_* = 3.12; surface-based: *M* = 0.57, *SD* = 0.10, *t*(21) = 3.23, *p* < 0.01, Cohen’s *d_z_* = 0.67]. An independent-sample *t* test was used to explore the roles of different types of features in incidental category learning. The result revealed that the accuracy in the edge-based condition was significantly higher than that in the surface-based condition, *t*(43) = 7.22, *p* < 0.001, Cohen’s *d* = 2.12. Thus, consistent with Experiment 1, the results confirmed that participants performed better in the edge-based condition than those in the surface-based condition.

#### Comparison Between Accuracies in Experiments 1 and 2

To compare the influence of the number of defined dimensions on the classification performance in incidental category learning, a 2 (condition: edge-based vs. surface-based) × 2 (the number of defined dimensions: 3 vs. 4) between-subject ANOVA was conducted on accuracy. The results revealed a significant main effect of condition, *F*(1, 80) = 65.23, *p* < 0.001, $\eta_{p}^{2}$ = 0.45 and a marginal significant interaction of condition by the number of defined dimensions, *F*(1, 80) = 3.70, *p* = 0.058, $\eta_{p}^{2}$ = 0.044. The main effect of the number of defined dimensions was not significant, *F*(1, 80) = 3.05, *p* = 0.085. The simple effect analysis revealed that the accuracy was higher when the number of defined dimensions was four in Experiment 2 than when it was three in Experiment 1 in the edge-based condition, *F*(1, 80) = 6.71, *p* < 0.05, $\eta_{p}^{2}$ = 0.08 but was not in the surface-based condition, *F*(1, 80) = 0.02, *p* > 0.80. The results suggested that increasing the defined dimensions can improve performance in the edge-based condition but not in the surface-based condition.

#### Categorization Proportions in the Testing Phase

Figure 5B shows the proportions of “yes” responses of different kinds of stimuli in the two conditions. As in Experiment 1, we analyzed the proportions of “yes” responses for different types of testing stimuli to examine whether the number of defined features influenced participants’ responses. A 2 (condition: edge-based vs. surface-based) × 4 (defined features: 1 vs. 2 vs. 3 vs. 4) mixed ANOVA conducted on the proportion of “yes” responses revealed a significant main effect of defined features, *F*(3, 129) = 146.82, *p* < 0.001, $\eta_{p}^{2}$ = 0.77, a significant effect of condition, *F*(1, 43) = 7.28, *p* < 0.05, $\eta_{p}^{2}$ = 0.15, and a significant interaction of condition by the defined features, *F*(3, 129) = 36.04, *p* < 0.001, $\eta_{p}^{2}$ = 0.46. The simple effect analysis revealed that when the number of defined features was one or two, the proportion of “yes” responses (i.e., the false alarm rate) was significantly lower in the edge-based condition than in the surface-based condition (both *p*s < 0.05); on the contrary, when there were four defined features, the proportion of “yes” responses (i.e., the hit rate) was significantly higher in the edge-based condition than in the surface-based condition [*F*(1, 43) = 11.08, *p* < 0.01, $\eta_{p}^{2}$ = 0.21]. That is, consistent with the results of Experiment 1, participants in the edge-based condition more correctly rejected non-category members and accepted category members than those in the surface-based condition. In addition, the proportion of “yes” responses significantly increased with the number of defined features in both conditions (both *p*s < 0.05), suggesting that participants learned to combine the features of four dimensions to classify the novel stimuli.

#### Probability Ratings

**Figure 6:**
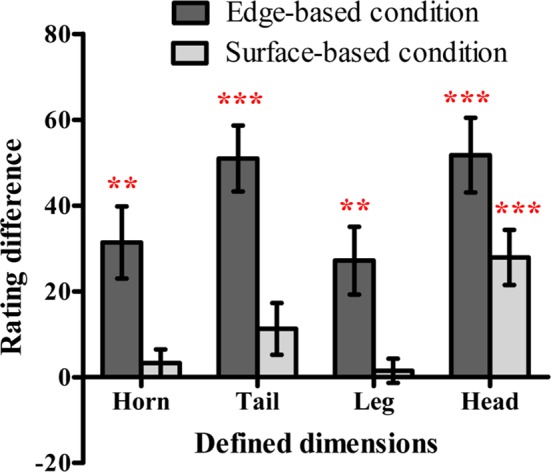
The difference scores of the probability rating for defined dimensions in the edge-based and surface-based conditions in Experiment 2. Error bars depict standard errors. *p < 0.05, **p < 0.01, ***p < 0.001.

To explore whether participants were aware of the relation between the defined features and the category membership, we also calculated the difference rating for each defined dimension as in Experiment 1 (see Figure 6). A one-sample *t* test revealed that in the edge-based condition, the difference ratings of the shape of the horn, tail, leg, and head were significantly above zero [horn shape: *t*(22) = 3.74, *p* < 0.01, Cohen’s *d_z_* = 0.78; tail shape: *t*(22) = 6.64, *p* < 0.001, Cohen’s *d_z_* = 1.38; leg shape: *t*(22) = 3.45, *p* < 0.01, Cohen’s *d*_z_ = 0.72; head shape: *t*(22) = 5.94, *p* < 0.001, Cohen’s *d_z_* = 1.24]. In the surface-based condition, the difference rating of the color of the head was significantly above zero [*t*(21) = 4.35, *p* < 0.001, Cohen’s *d_z_* = 0.95], while the difference ratings of the horn color, tail color, and leg texture were not (*p*s *>* 0.05). The results indicated that participants were partially aware of the relation between the defined features and the category membership.

As in Experiment 1, the accuracy was regressed on the significant rating differences of the defined dimensions. For the edge-based condition, the rating differences of the shape of the horn, tail, leg, and head did not predict the accuracy in the testing phase [*F*(4, 18) = 2.84, *p* > 0.05]. For the surface-based condition, the rating difference of head color also did not predict the accuracy in the testing phase [*F*(1, 20) = 3.42, *p* > 0.07].

### Discussion

Consistent with Experiment 1, the results of Experiment 2 showed that participants performed significantly better in the edge-based condition than in the surface-based condition when the defined dimensions increased to four, confirming that edge-based features play a more important role than surface-based features in incidental category learning. Interestingly, increasing the number of defined dimensions enhanced rather than decreased performance in the edge-based condition, while it did not significantly change performance in the surface-based condition. This further suggests that edge-based information and surface-based information play different roles in incidental category learning. The probability rating results suggest that people might be partially aware of the relation between the defined features and the category membership, but these awareness scores in the probability rating phase could not account for accuracy in the classification task in the testing phase.

## Experiment 3

In the first two experiments, each category was defined by a rule of three or four defined features and the category members presented in the training phase shared same features. However, in the typical prototype distortion task, some distortions were presented in the training phase, while the previous unseen prototypes and other distortions were presented in the testing phase. As no same features were shared by all stimuli in the training phase, it might be more difficult to learn the category defined by a similarity to the prototypes than by a rule. Thus, to increase the learning difficulty, the category members were defined by a similarity, which was manipulated through varying features in the five defined dimensions in Experiment 3. Specifically, the prototypes were created by defining the features of the five defined dimensions and then randomly changing the features of the other five non-defined dimensions; the different levels of distortions were created by remaining the non-defined features of the prototypes and then randomly changing the defined features of the prototypes. Thus, the prototypes and the distortions differed only in the defined features rather than the non-defined features. Consistent with the typical prototype distortion task, the stimuli with four defined features identical to the prototypes, i.e., low distortions, were presented during the training phase; the prototypes and the other distortions were presented during the testing phase. As in the previous research, there were no definitive criteria about which distortions can be classified as belonging to the category; we focused on the analysis of the proportions of “yes” responses to different distortions during the testing phase.

### Method

#### Participants

Thirty-two university students (16 females and 16 males) voluntarily participated in the experiment. They were randomly assigned to one of the two conditions. There were 16 (9 females; mean age = 21.50 years, *SD* = 2.85) in the edge-based condition and 16 (7 females; mean age = 21.06 years, *SD* = 2.46) in the surface-based condition. All participants reported normal or corrected to normal vision and were paid for their attendance. Two participants in the edge-based condition and one participant in the surface-based condition were excluded because their proportions of “yes” responses for the stimuli with high similarity were lower than the mean proportion minus two standard deviations.

#### Materials

The prototypes for the edge-based category were defined by the features of five edge-based dimensions and the features for the other surface-based dimensions could change randomly. Similarly, the prototypes for the surface-based category were defined by the features of five surface-based dimensions, and the features for the other edge-based dimensions could change randomly. Thus, there were a total of 32 prototypes in each category. Among them, two prototypes that have five defined features of the prototypes or non-prototypes in the other condition were excluded. As a result, there were 30 prototypes remained in each condition. We first created 60 stimuli by changing one defined feature of the remained 30 prototypes and maintaining the other features in the non-defined dimensions in each condition. Among them, 30 stimuli were presented during the training phase and the other 30 stimuli were presented during the testing phase. To create the stimuli with two, three, four, or five defined features different from the prototype in the testing phase, we changed one, two, three, or four defined features of the 30 stimuli that originally only have one defined feature changed, respectively. Thus, the stimuli in the testing phase differed only in the number of the defined features and all the non-defined features occurred with the same probability.

#### Design and Procedure

The design and procedure were similar to Experiment 1 except that there were 30 trials in the training phase, 180 trials in the testing phase, and 20 trials in the probability rating phase. Moreover, after the probability rating phase, there was an importance rating phase. The name of 10 dimensions was listed in a questionnaire, and participants were asked to rate how important each dimension was in their classification on a continuous scale from 0 to 100, where 0 = not important at all, 50 = moderately important, and 100 = very important.

### Results

There were no significant differences between the proportions of “yes” response for stimuli with zero and one defined features (both *p*s > 0.90), between the proportions of “yes” response for stimuli with two and three defined features (both *p*s > 0.20), and between the proportions of “yes” response for stimuli with four and five defined features (both *p*s > 0.90) in both conditions. Therefore, stimuli with zero and one defined feature of the prototypes were combined as the stimuli with low similarity; stimuli with two and three defined features of the prototypes were combined as the stimuli with medium similarity; stimuli with four and five defined features of the prototypes were combined as the stimuli with high similarity.

#### Categorization Proportions in the Testing Phase

**Figure 7:**
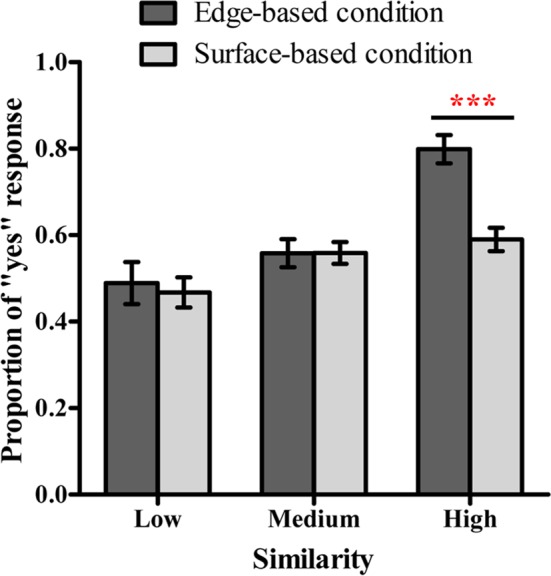
Proportion of “yes” responses for different types of stimuli in the edge-based and surface-based conditions in Experiment 3. Error bars depict standard errors. *p < 0.05, **p < 0.01, ***p < 0.001.

Figure 7 shows the proportions of “yes” responses of different types of stimuli in the two conditions. A one-sample *t* test was used in order to explore whether participants learned the category. In the edge-based condition, the proportion of “yes” responses was significantly above chance level (0.50) for stimuli with high similarity [*t*(13) = 9.10, *p* < 0.001, Cohen’s *d_z_* = 2.43] but not for stimuli with medium and low similarities (both *p*s > 0.08). In the surface-based condition, the proportion was significantly above chance level for stimuli with high and medium similarities [high: *t*(14) = 3.31, *p* < 0.01, Cohen’s *d_z_* = 0.86; medium: *t*(14) = 2.34, *p* < 0.05, Cohen’s *d_z_* = 0.60] but not for stimuli with low similarity (*p* > 0.10).

To examine whether the similarity to the prototype influenced performance in classification, a 2 (condition: edge-based vs. surface-based) × 3 (similarity: low vs. medium vs. high) mixed ANOVA on the proportion of “yes” responses was conducted. The results revealed a significant main effect of similarity, *F*(2, 54) = 26.98, *p* < 0.001, $\eta_{p}^{2}$ = 0.50, a significant main effect of condition, *F*(1, 27) = 5.11, *p* < 0.05, $\eta_{p}^{2}$ = 0.16, and a significant interaction between similarity and condition, *F*(2, 54) = 7.49, *p* < 0.01, $\eta_{p}^{2}$ = 0.22. The simple effect analysis showed that, in the edge-based condition, the proportion for stimuli with high similarity was significantly higher than the proportion for stimuli with medium and low similarities (both *p*s < 0.001). In the surface-based condition, however, the proportions for stimuli with high and medium similarities were significantly higher than that for stimuli with low similarity (both *p*s < 0.05). More importantly, for stimuli with high similarity, the proportion of “yes” responses was significantly higher in the edge-based condition than in the surface-based condition [*F*(1, 27) = 24.27, *p* < 0.001, $\eta_{p}^{2}$ = 0.47]. Therefore, participants from both groups learned the category representation and participants performed better in the edge-based condition than in the surface-based condition, which were consistent with the results in Experiments 1 and 2.

#### Probability Ratings in the Testing Phase

**Figure 8:**
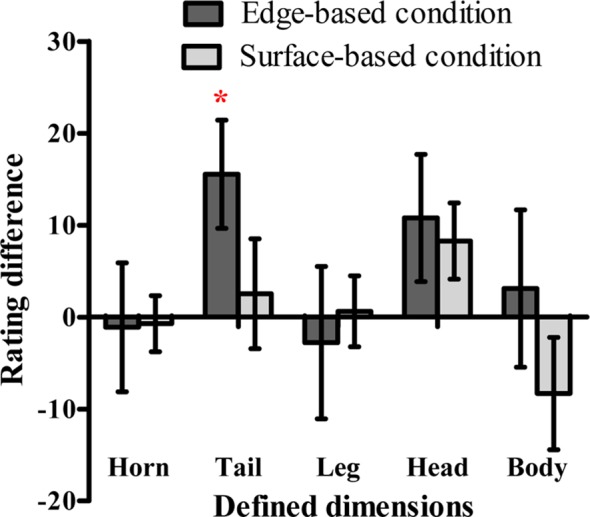
The difference scores of the probability rating for defined dimensions in the edge-based and surface-based conditions in Experiment 3. Error bars depict standard errors. *p < 0.05, **p < 0.01, ***p < 0.001.

To explore whether participants could be aware of the relation between the defined features and the category membership, we also calculated the difference rating for each defined dimension as in Experiment 1 (see Figure 8). A one-sample *t* test revealed that only the difference rating of the shape of the tail in the edge-based condition was significantly above zero [*t*(13) = 2.64, *p* < 0.05, Cohen’s *d_z_* = 0.70]. The results indicated that participants in edge-based condition might be only aware of the relation between the shape of the tail and the category membership.

As in Experiments 1 and 2, when the proportions of “yes” response for stimuli with high similarity was regressed on the rating difference of the shape of the tail in the edge-based condition, the results showed that the rating difference of the tail shape did not predict the proportions of “yes” response for stimuli with high similarity in the testing phase [*F*(1, 12) = 0.10, *p* = 0.76]. The results indicate that people mainly acquire implicit category knowledge.

#### Importance Rating

**Figure 9:**
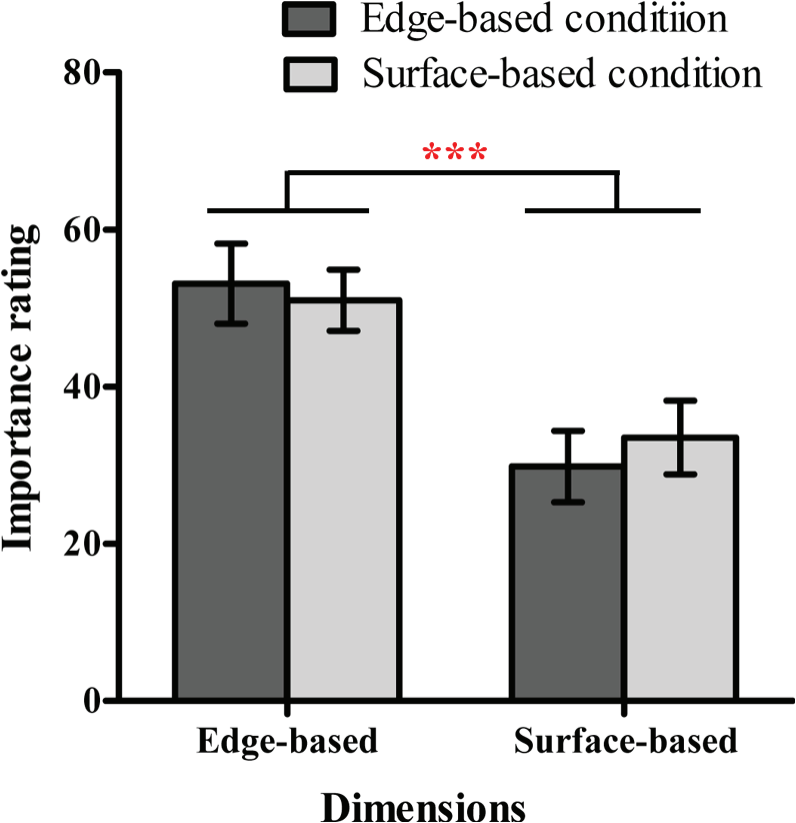
The importance ratings for the five edge-based dimensions and five surface-based dimensions in the edge-based and surface-based conditions in Experiment 3. Error bars depict standard errors. *p < 0.05, **p < 0.01, ***p < 0.001.

To explore whether participants were more reliant on edge-based or surface-based information in classification, we calculated the mean importance rating for the five edge-based and five surface-based dimensions separately in each condition (see Figure 9). A 2 (dimensions: edge-based vs. surface-based) × 2 (condition: edge-based vs. surface-based) mix ANOVA revealed only a significant main effect of dimensions [*F*(1, 27) = 26.47, *p* < 0.001, $\eta_{p}^{2}$ = 0.50]. The main effect of condition [*F*(1, 27) = 0.024, *p* = 0.88] and the interaction [*F*(1, 27) = 0.54, *p* = 0.47] did not reach significance. Therefore, regardless of whether the category was defined by edge-based or surface-based dimensions, participants always consider the edge-based dimensions as more important than the surfaced-based dimensions.

### Discussion

Consistent with the results of Experiments 1 and 2, the results of Experiment 3 showed that the proportion of “yes” response for stimuli with high similarity was higher in the edge-based condition than in the surface-based condition, confirming that there was a larger learning effect in the edge-based condition than in the surface-based condition. Interestingly, although participants in the edge-based condition were aware that the shape of the tail might be associated with the category membership, these awareness scores did not account for the performance in the testing phase, indicating that participants mainly acquire implicit category knowledge.

Moreover, although the category was defined by a similarity, participants might make classification based on one defined feature. If they did it in this way, the proportions of “yes” responses for stimuli with high similarity, medium similarity, and low similarity would be 0.90, 0.50, and 0.10, respectively. As the expected proportion for stimuli with medium similarity was equal to the chance level, we compared only the proportions for stimuli with high and low similarities with 0.90, and 0.10, respectively. One-sample *t* test revealed that the proportion for high similarity stimuli was significantly lower than 0.90 in both edge-based condition and surface-based condition (both *p*s < 0.01), while the proportions for stimuli with low similarity were significantly higher than 0.10 (both *p*s < 0.001). The results indicated that participants did not use one-single-defined-feature strategy during the testing phase.

## General Discussion

The purpose of this present study was to explore whether edge-based features play a more crucial role than surface-based features in incidental category learning. As expected, the results of Experiments 1 and 2 showed that when each category was defined by a rule, participants performed much better in the edge-based condition than in the surface-based condition, and increasing the number of defined dimensions enhanced rather than reduced the performance in the edge-based condition but not in the surface-based condition. When each category was defined by a similarity, the results of Experiment 3 also showed that participants could learn the two categories and that there was also a larger learning effect when the category was defined by edge-based dimensions than when defined by surface-based dimensions. The current study is the first to provide the convergent evidence that edge-based information is more important than surface-based information in the representation’s formation in incidental category learning.

Why is edge-based information more important than surface-based information in both object recognition and incidental category learning? One explanation is that edge-based features such as shape provide more salient holistic information compared with local information (Bell and Badcock, 2008; Bell et al., 2010; Osugi and Takeda, 2013), whereas surface-based features such as color and textures make it difficult to combine local parts to form a holistic representation. Thus, global precedence enables attention to be first directed to edge-based features, and the visual short-term memory might operate more efficiently on edge-based features than on surface-based features as a result (Alvarez and Cavanagh, 2008). Furthermore, edge-based information receives prior processing (Fu et al., 2016), and the human visual system might use schematic representations to encode and process object or scene categories (Walther et al., 2011).

Another explanation is from an evolutionary point of view: edge-based information such as shape is more stable than surface-based information such as color. For example, the colors of fruits change dramatically during growth, but their shapes do not. This might lead edge-based features to be more salient and important in object recognition and category learning. It has been found that infants use boundary information in object recognition earlier than they can use surface information. For example, four-month-old infants can use shape and boundary features to segment objects (Needham, 1999) and can use both color and shape information to individuate objects only by the age of 12 months (Tremoulet et al., 2000).

Interestingly, we found that increasing the number of defined dimensions enhanced rather than reduced performance in the edge-based condition but not in the surfaced-based condition. It may be because that increasing the number of defined dimensions makes the holistic processing of the perceptual representation easier to be learned in the edge-based condition but not in the surface-based condition. Importantly, not only was the category learning performance better in the edge-based condition than in the surface-based condition, but also the edge-based dimensions were rated as more important than the surface-based dimensions regardless of whether the category was defined by edge-based or surface-based dimensions, providing further convergent evidence that edge-based information plays a more important role in incidental category learning.

In our experiments, categories were defined by multidimensions and participants reported that they made their classification on multiple dimensions in Experiments 1 and 2. If they used rule-based strategies, the proportion of “yes” responses should be identical to the stimuli with defined features changed. However, our results showed that the proportion of “yes” responses increased with the number of defined features in Experiments 1 and 2. This was true in both edge-based and surface-based conditions, although it was more salient in the edge-based condition than in the surface-based condition. The results suggest that, although participant could combine all defined features in classification, they might use similarity-based strategy to make classifications. This was consistent with studies showing that incidental learning promotes similarity-based processing, whereas rule-based processing is involved in intentional learning (Wattenmaker, 1991; Love, 2002).

Finally, in our experiments, participants were asked to carefully observe each stimulus and rate how likeable it was in the training phase and were not asked to learn the category directly, and no trial-by-trial feedback was provided in both the training and the testing phases. This guaranteed that the learning process occurred incidentally. To examine whether participants were aware of the acquired knowledge, they were asked to report the probability that the stimuli including different features of the defined dimension belonged to one category. In Experiment 1, the rating results, which could account for the accuracy in the testing phase, indicated that participants were aware of the relation between the defined features and the category membership to some extent. Interestingly, with an increase in the defined dimensions from three to four in Experiment 2, these awareness scores in the probability rating phase could not account for accuracy in the classification task in the testing phase. Moreover, in Experiment 3, only participants in the edge-based condition were aware of the relation between one defined dimension and the category membership, but this awareness score also did not account for performance in the testing phase. This indicated that participants can acquire implicit category knowledge in incidental category learning.

To summarize, our results showed that people performed much better in the edge-based condition than in the surface-based condition regardless of whether a category is defined by a rule or a similarity. The results suggested that the category representation formed in incidental category learning might consist primarily of edge-based information rather than surface-based information, and future studies need to distinguish the different roles of different features in category learning.

## Author Contributions

XZ and QF designed the experiment. XZ performed the experiment and analyzed the collecting data. XZ, QF, MR, and YS wrote and revised the manuscript.

### Conflict of Interest Statement

The authors declare that the research was conducted in the absence of any commercial or financial relationships that could be construed as a potential conflict of interest.
